# Influence of Intra-Oral Scanner (I.O.S.) on The Marginal Accuracy of CAD/CAM Single Crowns

**DOI:** 10.3390/ijerph16040544

**Published:** 2019-02-14

**Authors:** Francesco Ferrini, Gianpaolo Sannino, Carlo Chiola, Paolo Capparé, Giorgio Gastaldi, Enrico Felice Gherlone

**Affiliations:** 1 Department of Dentistry, IRCCS San Raffaele Hospital and Dental School, Vita Salute University, 20123 Milan, Italy; ferrini.f@gmail.com (F.F.); paolocappare@gmail.com (P.C.); gastaldi.giorgio@hsr.it (G.G.); gherlone.enrico@hsr.it (E.F.G.); 2 Dental School, Vita Salute University, 20123 Milan, Italy; cchiola@me.com; 3 San Rocco Clinical Institute, 25050 Brescia, Italy

**Keywords:** CAD/CAM, digital impression, marginal accuracy, scanner, SEM

## Abstract

The aim of this in vitro study was to compare the quality of digital workflows generated by different scanners (Intra-oral digital scanners (I.O.S.s)) focusing on marginal fit analysis. A customized chrome-cobalt (Cr-Co) implant abutment simulating a maxillary right first molar was fixed in hemi-maxillary stone model and scanned by eight different I.O.S.s: Omnicam**^®^** (Denstply Sirona, Verona, Italy) CS3500**^®^**, CS3600**^®^**, (Carestream Dental, Atlanta, GA, USA), True Definition Scanner**^®^** (3M, St. Paul, MN, USA), DWIO**^®^** (Dental Wings, Montreal, Quebec, Canada), PlanScan**^®^** (Planmeca Oy, Helsinki, Finland), 3D PROGRESS Plus**^®^** (MHT, Verona, Italy), TRIOS 3**^®^** (3Shape, Copenhagen, Denmark). Nine scans were performed by each tested I.O.S. and 72 copings were designed using a dental computer-assisted-design/computer-assisted-manufacturing (CAD/CAM) software (exocad GmbH, Darmstadt, Germany). According to CAD data, zirconium dioxide (ZrO_2_) copings were digitally milled (Roland DWX-50, Irvine, CA, USA). Scanning electron microscope (SEM) direct vision allowed for marginal gap measurements in eight points for each specimen. Descriptive analysis was performed using mean, standard deviation, and median, while the Kruskal–Wallis test was performed to determine whether the marginal discrepancies were significantly different between each group (significance level *p* < 0.05). The overall mean marginal gap value and standard deviation were 53.45 ± 30.52 μm. The minimum mean value (40.04 ± 18.90 μm) was recorded by PlanScan^®^, then 3D PROGRESS Plus^®^ (40.20 ± 21.91 μm), True Definition Scanner**^®^** (40.82 ± 26.19 μm), CS3500**^®^** (54.82 ± 28.86 μm) CS3600^®^ (59,67 ± 28.72 μm), Omnicam^®^ (61.57 ± 38.59 μm), DWIO^®^ (62.49 ± 31.54 μm), while the maximum mean value (67.95 ± 30.41 μm) was recorded by TRIOS 3^®^. The Kruskal–Wallis tests revealed a statistically significant difference (*p*-value < 0.5) in the mean marginal gaps between copings produced by 3D PROGRESS Plus**^®^**, PlanScan, True Definition Scanner, and the other evaluated I.O.S.s. The use of an I.O.S. for digital impressions may be a viable alternative to analog techniques. Although in this in vitro study PlanScan**^®^**, 3D PROGRESS Plus**^®^** and True Definition Scanner**^®^** may have showed the best performances, all I.O.S.s tested could provide clinically encouraging results especially in terms of marginal accuracy, since mean marginal gap values were all within the clinically acceptable threshold of 120 μm.

## 1. Introduction

Digital dentistry is constantly growing since the first use of computer-aided design and computer-aided manufacturing (CAD/CAM) technology for prosthesis fabrication in 1980s [1]. Nowadays digital impression, including direct intraoral scanning or indirect digitization of casts derived from conventional impressions, can generate a stereolithography (STL) file which represents the first step of the digital path [2,3]. The clinical situation can be moved to a virtual ‘working cast-free’ environment. Nevertheless, when needed, physical casts can be still fabricated from the same STL files using rapid prototyping technologies.

Intra-oral digital scanners (I.O.S.s) have pushed dentistry into a full digital era, changing the daily routine both for dentists and technicians [4,5].

Performing intraoral scans may increase efficiency for several aspects. Impression trays and materials—which have to be dispensed, disinfected and then shipped to laboratory—are no longer required [6,7,8,9]. The electronic files can be digitally sent and stored saving time, cost, and space management. Distortion as well as volumetric variations related to impression material and die stone properties are eliminated while improving patient acceptance [6,7,8].

In addition to these important factors, the marginal accuracy—together with fracture stability and material biocompatibility—are still described as crucial for long-term success of restoration [10,11,12,13]. Precision and marginal accuracy must be carefully evaluated, since a marginal discrepancy could cause different negative occurrences: tooth sensitivity, luting seal loosing, periodontal disease, secondary caries, up to failure restoration [12,13,14,15,16]. To date, there is no evidence regarding a maximum marginal discrepancy that is clinically acceptable [17,18,19]. However, there is a consensus among several authors in accepting criterion established by McLean and von Fraunhofer (1971) who proposed a maximum marginal gap of 120 μm after a five-year examination of 1000 restoration gaps [20]. It should not be forgotten that the cement layer usually requires a space between 25 and 50 μm.

Since marginal fit is a fundamental factor for assessing the quality of a restoration, many studies, including literature reviews and systematic reviews, have addressed this issue [10,11,21,22,23,24,25,26,27]. The results show different values ranging from 3.7 to 206.3 μm, mainly due to difference in study designs, i.e., measurement method, sample size, quantity of measurements, restoration material, type of microscope, type of abutment, and finish line [10,11,28].

Over the last two decades, many I.O.S.s have been developed and successfully introduced, enhancing scanning speed and accuracy. Recent studies reported a comparable or even better general accuracy of the digital scans compared with the conventional impression methods [2,3,4,5,6,7,8,9].

The 3D data deriving from different intra-oral scanning systems, should not only be compared to the conventional impressions, but compared to each other too [29,30]. The aim of this in vitro study was to compare different I.O.S.s and evaluate—by SEM—marginal discrepancy of prosthetic restorations obtained with a full digital workflow.

## 2. Materials and Methods

**Figure 1:**
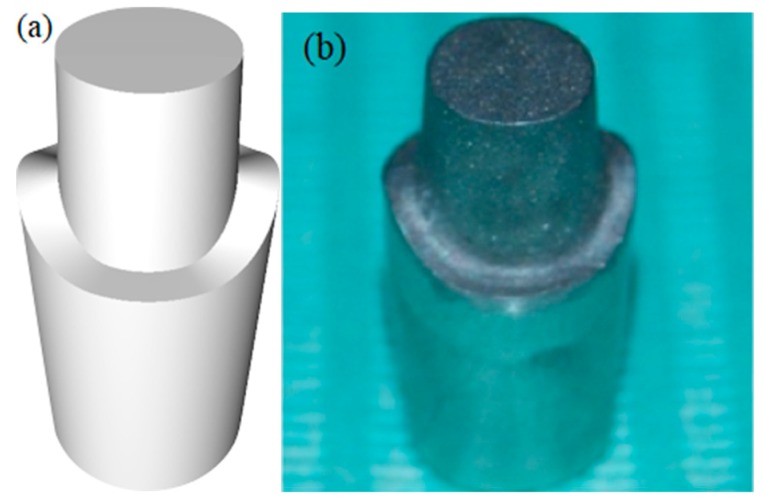
A customized Cr-Co implant abutment was manufactured according to the STL file generated by a 3D CAD modeling software, (**a**)3D CAD model; (**b**)Cr-Co milled abutment.

A customized Cr-Co implant abutment was manufactured according to the STL file generated by a 3D CAD modeling software (Rhinoceros 5, McNeel Europe, Barcelona, Spain) (Figure 1).

The model, simulating a maxillary right first molar, provided a diameter of 1 cm, height of 1 cm, a taper of 3°, and a 2 mm deep 360° chamfer as marginal design.

The finish line level was assumed to be similar to a natural tooth, i.e., it was more apical in the buccal and palatal area compared to interproximal regions, which were more coronal.

Sandblasting was performed to improve surface roughness and facilitate the scanning process.

Two vertical slots were made on the base of the abutment, as reference points, in order to allow the correct repositioning of the abutment in case of removal.

**Figure 2:**
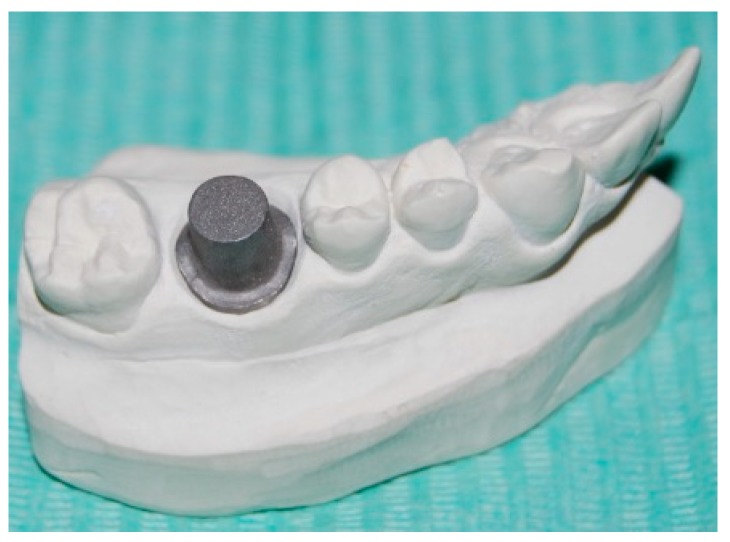
The abutment was then mounted and fixed in hemi-maxillary stone model.

The abutment was then mounted and fixed in hemi-maxillary stone model by using cyanoacrylate (Figure 2). The finish line was kept at the gingival level with respect to adjacent teeth.

The model was reduced in correspondence of the Co-Cr abutment to mimic the presence of gingival sulcus. A hole at the model bottom was made for an easy abutment removal.

Eight different I.O.S.s were used for the study: Omnicam**^®^** (Denstply Sirona, Verona, Italy) CS3500**^®^**, CS3600**^®^**, (Carestream Dental, Atlanta, GA, USA), True Definition Scanner**^®^** (3M, St. Paul, MN, USA), DWIO**^®^** (Dental Wings, Montreal, Quebec, Canada), PlanScan**^®^** (Planmeca Oy, Helsinki, Finland), 3D PROGRESS Plus**^®^** (MHT, Verona, Italy), TRIOS 3**^®^** (3Shape, Copenhagen, Denmark). Nine scans were performed with each tested I.O.S.

**Figure 3:**
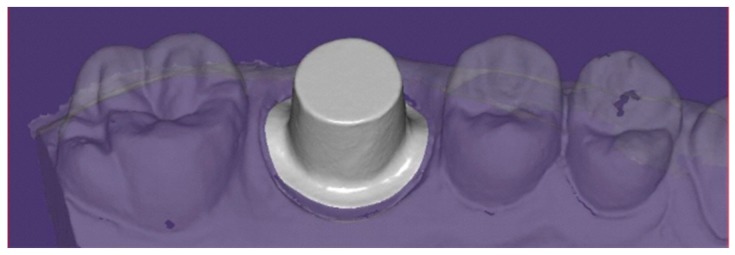
Scanned images were imported in Exocad CAD/CAM software and managed by a technician for core designing.

A total of 72 scans were managed by a technician for core designing using a dental CAD/CAM software (exocad GmbH, Darmstadt, Germany) (Figure 3).

**Figure 4:**
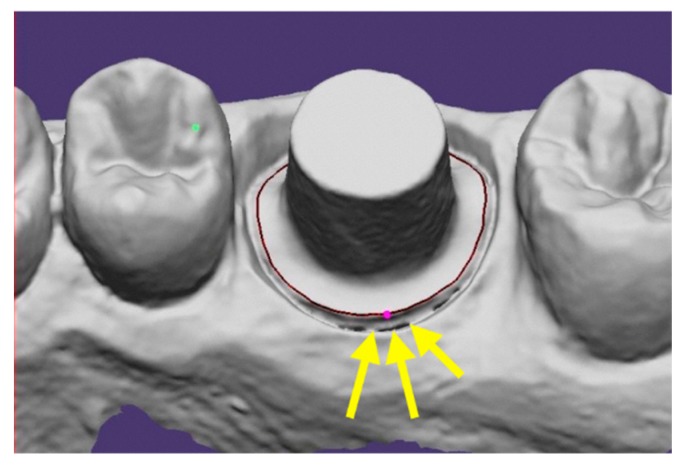
The finish line was automatically set by exocad software when clearly detectable, while in other cases manual adjustments were needed.

The luting space was set as 30 μm, while the core showed a uniform 1 mm thickness thanks the coping off-set function. Before starting the definitive coping production from each file, a test coping was produced in order to evaluate the right fit according to abutment geometry and prosthesis material. Once the right fit was found, the same parameters were set and kept unvariate for the whole production. The finish line was automatically set by Exocad software when clearly detectable, while in other cases manual adjustments were needed (Figure 4).

**Figure 5:**
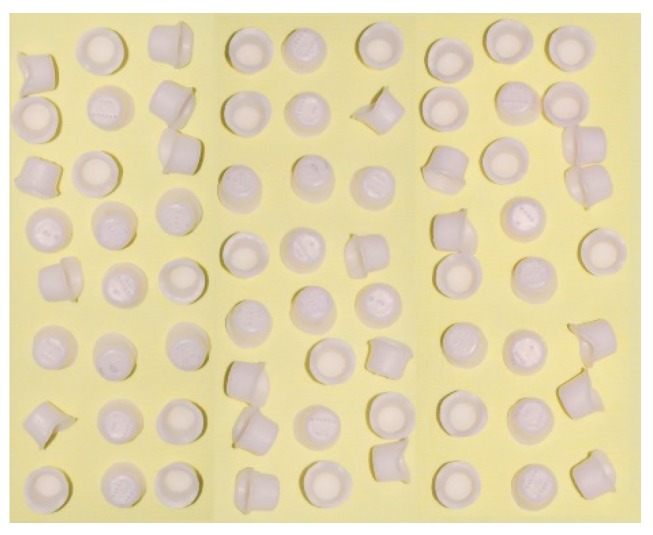
A total of 72 copings were milled according to test coping produced in order to evaluate the right fit.

CAD data were first sent to the CAM software (hyperDENT 8.2, Munich, Germany) and then to a five-axis milling machine (Roland DWX-50, Irvine, CA, USA). A high quality (H.Q.) milling program for all copings was selected and the time required for each coping to be milled was of 30 min (Figure 5).

Five disks of ZrO_2_ were used (Zircodent, ORODENT, Verona, Italia) and a new set of two burrs (DSF2-20, DSF1-15) was replaced for each disk.

Copings were then sintered for 10 h at 1500 °C in a ZIRKHONOFEN 600/V2 (Zirkozahn, Bolzan, Italy) special furnace. A fit check was performed for all the specimens and adjustments were performed in order to obtain a perfect coupling with the test abutment when needed.

A PhenomPro X Desktop scanning electron microscope (Thermo Fisher Scientific, Bothell, WA, USA) was used for SEM analysis. The incident electron beam of the SEM microscope was kept at a constant 25° inclination throughout the whole process thanks to the use of a metallic support for specimens. Photomicrographs were obtained from the center of each sample, with magnifications up to 410 X and then assessed (previously calibrated and blind to the experimental groups) by an examiner. The images were improved in terms of brightness and contrast in order to allow a perfect vision of the margins. The examiner accomplished three consecutive readings for each micrograph. The predominant score of the three readings was considered representative of the respective sample. Marginal gaps at the abutment–coping interface were measured at buccal, palatal, mesial, and distal aspects and at intermediate levels of the aforementioned points for a total of eight records. The marginal fit of the crown was defined as a mean value of the eight measurements for each coping.

Descriptive analysis was performed using mean, standard deviation, and median.

The Kruskal-Wallis test was performed to determine whether the marginal gaps were significantly different between each group. The significance level was set at *p* < 0.05. Statistical calculations were performed with the statistical software SPSS 14 for Windows (SPSS, IBM, Armonk, NY, USA).

## 3. Results

The SEM analysis of marginal gap performed in 576 points—i.e., 8 for each coping—showed a total mean value of 53.45 μm (SD 30.52, Median 50).

**Figure 6:**
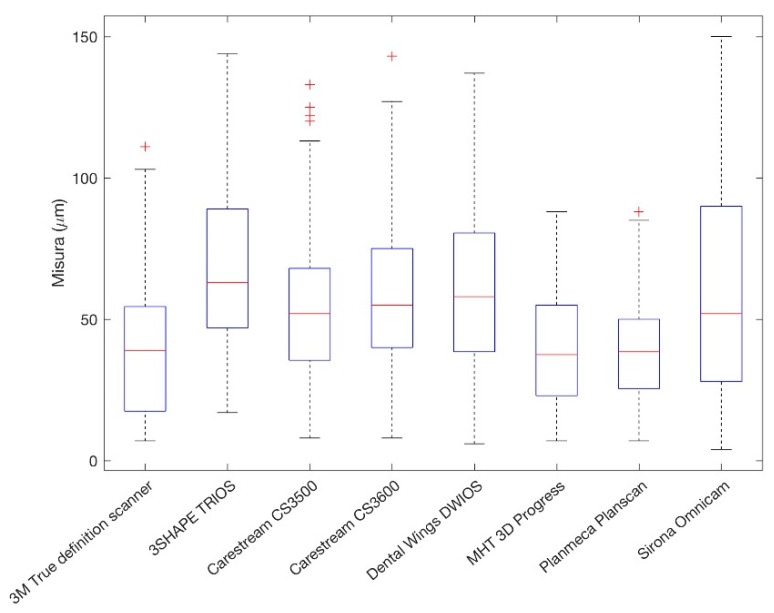
Box-plot representing the sample distribution. Outlier values are indicated by “+” symbols.

Mean, standard deviation, and median of the marginal gap values measured at abutment—coping interface of the eight groups are described in Table 1. Sample distribution is represented by the box-plot in Figure 6.

**Figure 7:**
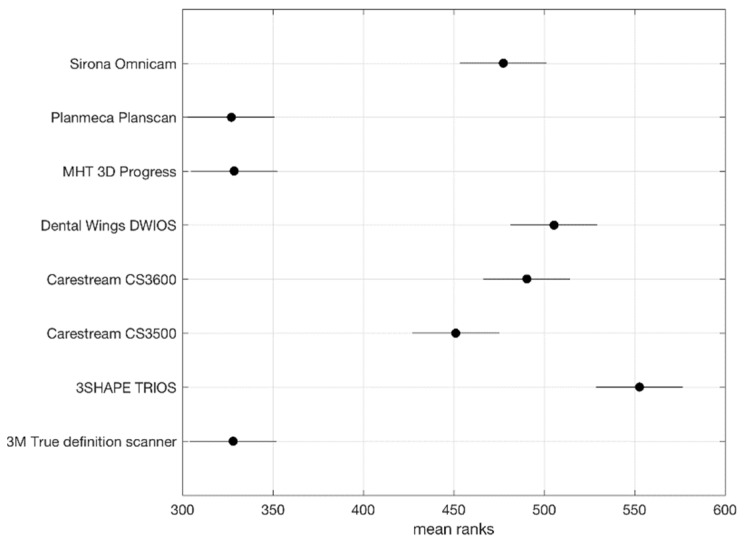
The Kruskal-Wallis tests revealed a statistically significant difference (*p*-Value < 0.5) in the mean marginal gaps between copings produced by different I.O.S.s.

The minimum mean value was 40.04 μm (SD 18.90, Median 38.50) and was recorded by PlanScan**^®^**, while the maximum mean value of 67.95 μm (SD 30.41, Median 63) was recorded by TRIOS 3**^®^**. Eighty-one measurements were higher than 100 μm. The Kruskal-Wallis tests revealed a statistically significant difference (*p*-Value < 0.5) in the mean marginal gaps between copings produced by 3D PROGRESS Plus**^®^**, PlanScan**^®^**, True Definition Scanner**^®^**, and the other evaluated I.O.S.s (Figure 7).

## 4. Discussion

The purpose of this in vitro study was to evaluate marginal precision in single restorations produced by a full digital line. The standardization process provided the I.O.S. used to perform digital impressions, as the unique variable. Hence, the first limitation of the present in vitro study may be related to each proprietary file, whose conversion into STL file, for the CAD management, could have led to a loss of quality and affected the whole workflow.

In accordance with protocols adopted by other authors for marginal gap evaluation, the same model was used for all the tests. [17,20,23] A customized Cr-Co implant abutment, featuring a tooth-like shape, was used for all the impressions. The use of a metal die, as the single standard master die, ensured standardized conditions for both impressions and following discrepancy analysis. Moreover, a steel model allowed for easy cleaning procedures without risk of surface damage [31]. It should not be underestimated that a metallic surface, although sanblasted, was not the ideal one to be digitally scanned, but all the scanners were used strictly according to with manufacturer guidelines. In this study, scanning systems (DWIO**^®^** and True Definition Scanners**^®^**), which required the use of an opaque powder coating of titanium dioxide for producing uniform light dispersion and increasing scan accuracy [9], were tested last in order to avoid even the slightest variation on the abutment during cleaning.

The abutment shape was intended to be similar to a natural tooth and the finish line as well, i.e., it was more apical in the buccal and palatal area compared to interproximal regions. The model was reduced in correspondence of the abutment to mimic the presence of the gingival sulcus and a clearly detectable juxta-gingival finish line was simulated. The finish line drawing was automatically performed by mean Exocad CAD/CAM software, except for True Definition Scanner**^®^** data, which required a manual detection. In the present study, a juxta-gingival ideal positioning of the finish line may have postively affected True Definition Scanner**^®^** overall accuracy, as comfirmed by the mean marginal gap value (40.82 ± 26.19 μm). These findings could partially agree with Nedelcu et al. [32], who analyzed, among other parameters, the level of finish line distinctness (FLD) and finish line accuracy (FLA), in seven I.O.S.s and one conventional impression (IMPR). In that study, True Definition Scanner**^®^**, together with DWIO**^®^** and Planscan**^®^**, showed low overall FLD and low FLA in subgingival areas compared to other I.O.S.s and IMPR.

Many authors have performed marginal fit evaluations by sectioning [27,33] the specimens and measuring it. In the present study, a non-destructive quantification was performed by SEM direct vision in order to reduce potential distortions generated by cutting and to perform multiple measurements. [29,34] The use of a customized support for the specimens allowed the examiner to achieve accuracy and repeatability of the measurements due to a constant 25° inclination of the incident electron beam with respect to evaluated surfaces. Although a section would allow for internal gap evaluation, only external marginal gap measurements—i.e., excluding internal axial and occlusal gaps—were performed by means SEM analysis. According to recent findings of Dauti et al., [35] external marginal gaps evaluation was sufficiently accurate in determining prosthesis precision. In a recent paper, Rodiger et al. [36] evaluated the influence of different materials on the marginal accuracy of CAD/CAM-fabricated crown copings using a lab-based scanner and a non-destructive measurement by light microscope. The authors found significantly increased mean and averaged maximum marginal gap values for YSZ copings compared to the CoCr and titanium copings, highlighting the material influence. In the present study, only ZrO_2_ copings were used in order to reduce variables. The mean marginal gap values (53.45 ± 30.52 μm) similar to those found by Rodiger et al. which ranged from 46.92 ± 23.12 μm (titanium) to 68.25 ± 28.54 μm (YSZ) may further comfirm a comparable or even better general accuracy of the digital scans compared with the conventional impression methods [2,3,4,5,6,7,8,9].

The marginal accuracy is one of the most important factors affecting long-term success in fixed restorations. A precise value of an acceptable marginal discrepancy has not yet been defined [17,18,19], however many authors agree in recognising a marginal gap less than 120 μm as clinically reasonable. [20] In this study, all mean values of marginal gap were below 120 μm regardless of the I.O.S. used. It should be underlined that the luting space was set as 30 μm; however, copings were not fixed on the abutment in order to use the same abutment for all the tests. Results could have been affected by the lack of the cement layer.

An experimental study evaluating and comparing full-arch scanning precision of conventional and digital impressions concluded that accuracy of digital impressions was similar to that of conventional impressions, probably due to a powder coat spraying [8]. Powder thickness may reduce scanning accuracy although scanner algorithm already provided the powder presence [31].

All the I.O.S.s tested in this study could allow for prosthesis fabrication featuring satisfactory marginal gap values, since the total mean was 53.45 ± 30.52 μm.

PlanScan**^®^**, 3D PROGRESS Plus**^®^**, and True Definition Scanner**^®^** showed highly positive results with mean values ranging from 40.04 to 40.82 μm.

In contrast the highest mean value, i.e., 67.95 ± 30.41 μm, was recorded for Trios 3.

Accuracy was confirmed by the S.D. values, which ranged from 18.90 to 26.19 for the aforementioned three scanners and from 28.86 to 31.52 for the others scanners, except for Omnicam**^®^** (S.D. 38.59). A low standard deviation may be mainly due to the repeatability in scanning results, which highlights the quality of the device. The repeatability of an intraoral scanning could represent a key factor, since the intraoral scanning is affected by the handling of the operator. Both movements of the patient and the scanner could favor larger discrepancy compared to extraoral scanning.

Guth et al. [37] evaluated repeated scans simulating direct intraoral and indirect extraoral scanning. The authors concluded that the reproducibility of extraoral scanning was better than that of intraoral scanning showing a systematic error of 13 μm and 5 μm, respectively. These findings were further confirmed by other authors [35] who reported a 20 μm or less systematic error in extraoral scanning. Even if a wide marginal gap range was found, all the tested I.O.S.s could allow to achieve accuracy levels below the threshold value proposed by McLean and von Fraunhofer. [20]

Statistical analysis performed by Kruskal–Wallis tests furthermore highlighted the better performances for PlanScan**^®^**, 3D PROGRESS Plus**^®^**, True Definition Scanner**^®^**. A statistically significant difference (*p*-value < 0.5) in the mean marginal gaps between copings produced according impression from PlanScan**^®^**, 3D PROGRESS Plus**^®^**, True Definition Scanner**^®^**, and the other evaluated I.O.S.s was found.

It should not be forgotten that, the standardization process of this in vitro study could have affect the results, because far from the real clinical conditions. The I.O.S. intraoral behavior would be different since dental tissue, with surrounding gingival tissues, has a totally different light response compared with a metal abutment as provided in this study.

## 5. Conclusions

Within the limitations of this study, it is possible conclude that the use of I.O.S. for digital impressions may be a viable alternative to analogical technique. The reliability of the workflow in term of marginal accuracy is enhanced by all the advantages lying into a full digital environment.

Although in this in vitro study PlanScan**^®^**, 3D PROGRESS Plus**^®^**, True Definition Scanner**^®^** may have showed best performances, all I.O.S.s tested could provide clinically encouraging results especially in terms of marginal accuracy. More long-term in vivo studies are needed to confirm the effectiveness of a full digital line in prosthetic rehabilitation, since the limitations of this in vitro study could have affect the results.

## Figures and Tables

**Table 1:** Mean, standard deviation and median of the marginal gap values measured at abutment—coping interface of the eight groups. Omnicam**^®^** (Denstply Sirona, Verona, Italy) CS3500**^®^**, CS3600**^®^**, (Carestream Dental, Atlanta, GA, USA), True Definition Scanner**^®^** (3M, St. Paul, MN, USA), DWIO**^®^** (Dental Wings, Montreal, Quebec, Canada), PlanScan**^®^** (Planmeca Oy, Helsinki, Finland), 3D PROGRESS Plus**^®^** (MHT, Verona, Italy), TRIOS 3**^®^** (3Shape, Copenhagen, Denmark).

Scanner	Mean	SD	Median
PlanScan^®^-Planmeca	40.04	18.90	38.50
3D PROGRESS Plus^®^-MHT	40.20	21.91	37.50
True Definition Scanner^®^-3M	40.82	26.19	39
CS3500^®^-Carestream Dental	54.82	28.86	52
CS3600^®^-Carestream Dental	59.67	28.72	55
Omnicam^®^-Denstply Sirona	61.57	38.59	52
DWIO^®^-Dental Wings	62.49	31.54	58
TRIOS 3^®^-3Shape	67.95	30.41	63
Total	53.45	30.52	50
